# Alcohol Misuse among University Staff: A Cross-Sectional Study

**DOI:** 10.1371/journal.pone.0098134

**Published:** 2014-07-29

**Authors:** Susanna Awoliyi, David Ball, Norman Parkinson, Victor R. Preedy

**Affiliations:** 1 Department of Primary Care and Public Health Sciences, King's College London, London, United Kingdom; 2 Department of Nutrition and Dietetics, King's College London, London, United Kingdom; 3 Institute of Psychiatry, King's College London, London, United Kingdom; 4 Diabetes and Nutritional Sciences Division, King's College London, London, United Kingdom; Penn State College of Medicine, United States of America

## Abstract

**Objectives:**

To examine the prevalence of hazardous drinking among staff in a UK university and its association with key socio-demographic features.

**Design:**

A cross-sectional study.

**Setting:**

A university in the UK.

**Participants:**

All employees on the university employee database were eligible to participate. Those who completed and returned the questionnaire were included in the sample. Respondents were 131 university employees.

**Primary and Secondary Outcome Measures:**

An AUDIT cut-off score of ≥8 was used as a measure of hazardous drinking. AUDIT total score as well as a score of ≥1 in each of the three conceptual domains of alcohol consumption (questions 1–3), dependence symptoms (questions 4–6) and alcohol-related problems (questions 7–10) were used as indicators of levels of drinking and alcohol-related consequences. Secondary outcomes were employees' demographics.

**Results:**

Over one third (35%) of respondents were classified as hazardous drinkers. Twenty three per cent reported having blackouts after drinking and 14% had injuries or had injured someone. The odds of being a hazardous drinker for an employee in central departments (Human Resources, Registry etc) is only one third of that of an employee in science and health-related departments (OR = 0.35, 95% CI = 0.14 to 0.91). The proportion of hazardous drinkers was higher in males compared to females (43% and 30% respectively), part-time compared to full-time (46% and 34% respectively), and academic compared to non-academic employees (39% and 32% respectively), although these were not statistically significant (p>0.05). Furthermore, age, religion and ethnic origin were not found to be significantly associated with hazardous drinking, although total scores were significantly lower for ethnic minorities compared to white employees (p = 0.019).

**Conclusions:**

In this study, hazardous drinking was highly prevalent among university employees. However, overt recruiting of staff to address sensitive issues such as alcohol misuse is problematic.

## Introduction

Alcohol misuse is a major public health issue, costing the UK economy up to £25.1 billion pounds per year [1]. In the UK, alcohol is one of the top three lifestyle risk factors for morbidity and mortality after smoking and obesity [2]. Alcohol consumption is related to over 60 different medical conditions [3], as well as injuries, violent offences and lost productivity at work. This places a huge burden on health services, costing the NHS £2.7 billion each year [4]. In 2009/10, there were over 1 million alcohol-related hospital admissions, more than double that in 2002/03 [5], and it is projected to rise to around 1.5 million by 2014/15 [6]. Similarly, deaths directly attributable to alcohol increased by 20% between 2001 and 2009 [5]. It is estimated that up to 22,000 premature deaths a year are alcohol-related [7]. In 2010/11, almost 1 million violent crimes were alcohol-related [8].

A major theme of the UK Government's Alcohol Harm Reduction Strategy is ‘better identification and treatment of alcohol problems'. A number of screening questionnaires have been developed to identify excessive drinking, of which the Alcohol Use Disorders Identification Test (AUDIT) is regarded as the gold standard [7]. The AUDIT is an alcohol screening tool that focuses on alcohol consumption and related problems. Developed by the World Health Organisation to detect hazardous drinking, a pattern of alcohol consumption that increases the risk of harmful consequences for the user [9], the AUDIT was validated cross-nationally within primary health care settings in six countries [10].

Around 70 per cent of people who have a drinking problem are in employment [11]. The current economic climate and lack of job security may have exacerbated the problem [12]. Conversely, some studies suggest that alcohol-related problems and mortality are less prevalent among those who are working [13], indicating that work could be a protective factor against alcohol misuse and dependence. There is evidence that employee alcohol use is prevalent enough to warrant further research into their causes and effects [14]. The impact of hazardous drinking may go beyond harm to employees' health and the organisation's productivity and reputation; members of the public may also be put at risk. For example, Hermansson *et al* reported that around 18 per cent of employees in a large workplace within the transport sector screened positive on the AUDIT for “elevated and risky” drinking [15]. This proportion may be deemed cause for alarm given the ‘safety sensitive’ nature of the industry. Nearly a third of police officers reported hazardous levels of alcohol consumption [16]. As well as highlighting the importance of research into alcohol use at work, these studies demonstrate the usefulness and feasibility of the AUDIT as a workplace screening tool.

In the university setting, there is a very large body of research evidence that shows high levels of alcohol consumption and related harm in the student population in the UK [17] and USA [18]. By contrast, little is known about the extent or distribution of alcohol use in university employees either in the UK or other countries. This study aimed to bridge this gap by presenting unique, quantitative data on the prevalence of hazardous drinking among employees at a higher education institution in the UK. The main objective of this study was to investigate the prevalence of hazardous drinking in university employees. A secondary objective was to investigate associations between hazardous drinking and key socio-demographic characteristics.

## Methods

### Study design and participants

A descriptive cross-sectional survey was conducted among staff at a UK university. All staff employed by the university were eligible for this study. Participants were therefore recruited via a circular email to all employees and those who responded positively were sent an information sheet and questionnaire, after piloting. The questionnaire consisted of the AUDIT and questions about socio-demographic characteristics, namely gender, age, department, employment, job, ethnicity and religion. The method of categorization was obtained from Human Resources and the responses of the participants. Participants answered the questions voluntarily and anonymously. In addition to returning the questionnaire by internal post (instead of email), age groups were used instead of age. Profession was not asked in order to maintain anonymity.

This study had the approval of King's College London Research Ethics Committee. Participants were sent information sheets along with the Questionnaire, which clearly stated in writing that returning the questionnaire would be taken to mean consent and that it would not be possible for participants to withdraw their data once they have submitted it because the questionnaire was anonymous. Permission was not obtained by Human Resources to send questionnaires to all employees indiscriminately, due to the sensitive nature of the subject matter.

### Measurement of alcohol use

The original 10-item AUDIT questionnaire [19] was used to estimate prevalence of hazardous drinking, defined as the proportion of participants scoring 8 or above on the AUDIT. Although, in some cases, lowering or raising the cut-off points may improve detection [20], a cut-off score of ≥8 was found to be a “reasonable approximation to the optimal for a variety of endpoints” [21]. In the development of the AUDIT, a total score of eight or above was recommended as an indicator of hazardous or harmful use [10]. Furthermore, in national surveys, an AUDIT score of 8 or more was used as an indicator of hazardous drinking [22].

Each question was scored from zero to four (questions 1–8: scored either 0, 1, 2, 3 or 4; questions 9 and 10: scored either 0, 2 or 4). The total score can range from zero to 40. Furthermore, the AUDIT can further be divided into three conceptual domains – alcohol consumption (questions 1–3), dependence symptoms (questions 4–6) and alcohol-related problems (questions 7–10). These domains can be used for a more specific interpretation of a participant's total score as follows: A score of 1 or more on questions 2 or 3 is indicative of hazardous alcohol consumption. A score above 0 on questions 4–6 may imply the presence or beginning of alcohol dependence, and on questions 7–10 that alcohol-related harm is already being experienced [10]. In this paper we use hazardous as shorthand for both those at risk of harm and those already experiencing it.

### Statistical methods

There were 5,370 employees at the university at the time of the study: 2,516 (47%) men and 2,854 (53%) women. Sample size was found from a one-sample binomial test using the statistical package Stata (version 6.0). Using a null hypothesis that five per cent of employees misuse alcohol and an alternative hypothesis of 10 per cent (both false in the results), at 90 per cent statistical power, a sample size of 264 was calculated. This null hypothesis was used as the National Institute for Health and Clinical Excellence (NICE) public health guidance 24 implies there is a high prevalence of hazardous drinking if it is greater than 5% [23].

Fewer questionnaires than the calculated sample size were sent out. This is because of the serious nature of addressing substance misuse, albeit alcohol, in the workplace. Although a level of confidentiality was assured, participants and all recipients of the email circular had no way of verifying this. Furthermore the number of distributed questionnaires was 171 and this number does not invalidate the results, but it should be clearly noted as a limitation. Various studies on substance misuse and even non-substance misuse using questionnaires have reported numbers of distributed questionnaires ranging from 67–125 [24]–[27]. Thus, 125 questionnaires were used in analysis of diabetes services [24], 120 in ophthalmic research [25], 110 attendees at an alcohol treatment facility [26], which used the AUDIT, and in another study using the AUDIT as low as 67 questionnaires [27]. Response rates have ranged from 74% to 100%.

Although the specific numbers of men and women needed for the sample to be representative of the numbers in the university population was 124 (47%) and 140 (53%), respectively, we were mindful of the fact that participation would be potentially low due to the sensitive nature of volunteering information to authorities that may indicate alcohol misuse. Therefore, all members of employed staff who agreed to participate were included in the sample to increase coverage for socio-demographic characteristics. Participants were emailed on an initial anonymous basis without prior knowledge of gender.

However it must be emphasised that whilst sample size may predict an ideal participation ratio, consideration also needs to be given to the fact that employees may find such questionnaires intrusive and/or suspicious. Such negative traits would perhaps result in a lower level of respondents. Furthermore, we argued that to make repeated requests to obtain a high ratio of respondents could have been considered as harassment.

The questionnaire data were verified by double entry using the statistical programme EpiData (version 3.1). Analyses were performed using the statistical software package SPSS (version 15.0) and Microsoft Excel 2007 used for some graphs. A one-sample binomial test was used to test whether the results were significantly different from the null hypothesis. AUDIT total score was also transformed into a new binary variable: 0 for employees who scored <8 and were at low risk of harmful consequences (including abstainers) and 1 for those scoring 8+ and were classified as hazardous drinkers at risk of harmful consequences. Logistic regression was used both univariately to test for associations between drinking status (low risk or hazardous) and socio-demographic variables and multivariately to examine several variables simultaneously. Associations between drinking status and socio-demographic characteristics were evaluated by odds ratios and 95% confidence intervals obtained from logistic regression. Given that some of the demographic categories are small (e.g. n = 3 for the under 25 age group), it was necessary to recode some of the variables into fewer categories for the analysis.

We also examined socio-demographic differences in the AUDIT total score because a low total score may indicate low alcohol consumption and few alcohol-related consequences whereas a high score indicates high consumption and severe consequences [28]. Associations between total score and socio-demographic characteristics were analysed using Mann-Whitney and Kruskal-Wallis tests. A p value of <0.05 was taken to be statistically significant for all statistical tests.

## Results

Out of the 171 questionnaires that were sent out, 131 were received by the closing date, giving a response rate of 77 per cent. This response rate is similar to that of hospital consultants (73%) [29], academic employees (72%) [28], and better than police personnel (67%) [16], a highly educated workforce (60%) [30] and employees of a higher education institution (38%) [31]. Some respondents did not answer all questions as reported in **Table 1**. The total number of fully completed questionnaires was 116. Two respondents did not complete the AUDIT but gave sufficient information to confirm an overall score of <8 and as such were coded within the data set, while their total score was coded as missing. Two respondents did not report their gender and therefore their gender was coded as missing.

**Table 1:** Demographic characteristics of respondents.

Demographic Characteristics	N
**Gender**	
Male	53 (41%)
Female	76 (59%)
*Total*	*129*
**Age group**	
Under 25	3 (2%)
25–29	20 (15%)
30–39	34 (26%)
40–49	35 (27%)
50–59	25 (19%)
60 and over	13 (10%)
*Total*	*130*
**Ethnicity**	
British/white	98 (76%)
Irish/white	5 (4%)
Other white background	19 (15%)
White and black Caribbean	1 (1%)
White and black African	1 (1%)
White and Hispanic/mixed race	1 (1%)
Irani/Asian	1 (1%)
Caribbean/black	2 (2%)
African/black	1 (1%)
*Total*	*129*
**Religion**	
None	58 (49%)
Christian	52 (44%)
Jewish	3 (3%)
Muslim	1 (1%)
Other	5 (4%)
*Total*	*119*

A total of 131 employees responded to the Questionnaire though not all answered every question. Figures for Total are given for each question. Other white background – respondents replied European, Irish Hispanic, Australian, Jewish American, French, Dutch, Portuguese, South American and Greek. Religion other includes Pagan and Zoroastrian.

### The distribution of socio-demographic characteristics within the sample

The percentage of respondents who described themselves by each category of the demographic characteristics is shown in **Tables 1** **and** **2**. The sample included more women (59%) than men (41%), which reflects the gender distribution (53% and 47%, respectively) in the university at the time of the study. The sample included a higher percentage of respondents aged 30 to 39 (26%) and aged 40 to 49 (27%) than other age groups. There was a similar distribution, i.e. respondents aged 30–39 (32%) and aged 40–49 (25%), in the university population.

**Table 2:** Demographic characteristics of respondents.

Demographic Characteristics	N
**Department**	
Central departments	33 (26%)
Life sciences	26 (20%)
Dentistry	5 (4%)
Humanities	4 (3%)
Psychiatry	5 (4%)
Law	4 (3%)
Medicine	21 (16%)
Nursing	13 (10%)
Physical sciences	6 (5%)
Social sciences	12 (9%)
*Total*	*129*
**Employment**	
Fixed-term Full-time	35 (27%)
Fixed-term Part-time	8 (6%)
Flexible Full-time	1 (1%)
Flexible Part-time	2 (2%)
Regular Full-time	76 (59%)
Regular Part-time	8 (6%)
*Total*	*130*
**Job**	
Administrative	46 (35%)
Manual	3 (2%)
Research	21 (16%)
Secretarial/clerical	7 (5%)
Teaching	17 (13%)
Teaching and research	29 (22%)
Technical	7 (5%)
*Total*	*130*

A total of 131 employees responded to the Questionnaire though not all answered every question. Figures for Total are given for each question. Other white background – respondents replied European, Irish Hispanic, Australian, Jewish American, French, Dutch, Portuguese, South American and Greek. Religion other includes Pagan and Zoroastrian.

The respondents were predominantly white (95%), with the majority (76%) being British. This should be considered in the context of the ethnic breakdown in the university under study in which the majority of employees are white (76%), with 53 per cent being British. There was a higher representation of agnostics (49%) and Christians (all denominations; 44%) than other religious groups in the sample. There were no comparative data for the university population, from which the sample was drawn, on religious affiliation.

The respondents were mostly academic staff, i.e. teaching and research (22%), research (16%) and teaching (13%), and administrative staff (35%). The single largest department category was Central Departments (26%). It was followed by Life Sciences (20%) and Medicine (16%). Most respondents reported their employment as regular full-time (59%) and fixed-term full-time (27%), which reflects the composition (regular full-time (51%) and fixed-term full-time (25%) in the university under study.

### Prevalence of hazardous drinking

**Figure 1:**
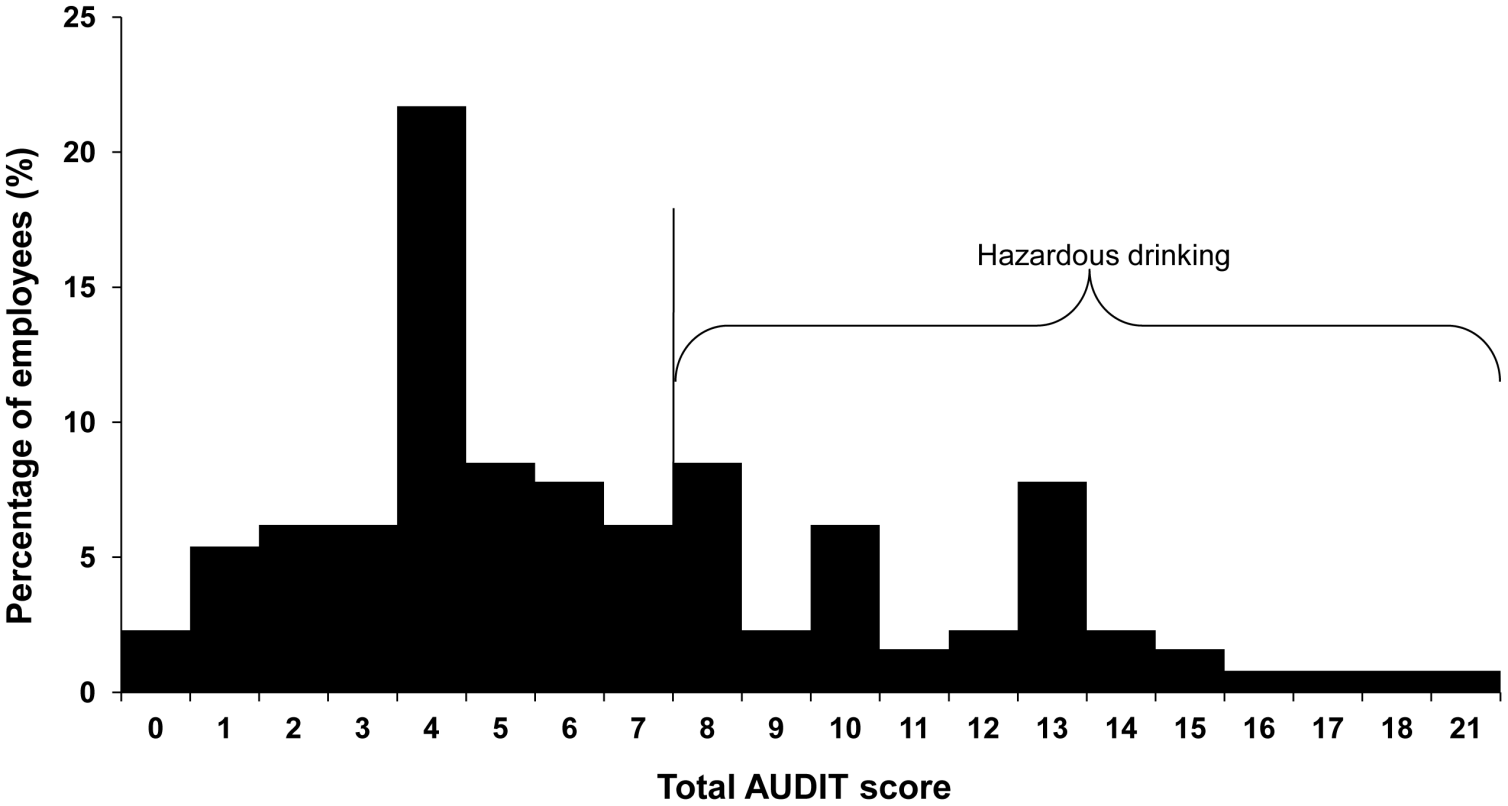
Distribution of total AUDIT score among respondents. Columns represent the percentage of respondents achieving each total AUDIT score (n = 129).

The proportion of respondents with an AUDIT score ≥8 was found to be 35 per cent. Consequently, the null hypothesis was rejected (p<0.001 using a one-sample binomial test). Three respondents (2%) were teetotallers (2 women and 1 man; **Figure 1**). Twenty three men (43%) and 23 women (30%) were classified as hazardous drinkers. The percentage of hazardous drinkers with respect to all socio-demographic characteristics is displayed in **Table 3**.

**Table 3:** Association between drinking status and socio-demographic characteristics.

Characteristics^(a)^	<8 (n = 85)		8+ (n = 46)		Unadjusted p value	OR	95% CI for OR	
	N	%	N	%			Lower	Higher
**Gender**					**0.127**			
Female	53	70	23	30		reference category		
Male	30	57	23	43	-	1.767	0.850	3.670
**Age group**					**0.554**			
Under 30	13	57	10	44	0.350	1.667	0.571	4.865
30–39	20	59	14	41	0.398	1.517	0.577	3.987
40–49	25	71	10	29	0.780	0.867	0.318	2.363
50+	26	68	12	32		reference category		
**Department**					**0.078**			
Science and health-related	43	57	33	43		reference category		
Arts	14	70	6	30	0.281	0.558	0.194	1.609
Central departments	26	79	7	21	0.031	0.351	0.136	0.907
**Employment**					**0.389**			
Full-time	74	66	38	34		reference category		
Part-time	10	56	8	44	-	1.558	0.568	4.271
**Ethnicity**					**0.998**			
British	62	63	36	37		reference category		
Other white	15	63	9	38	0.944	1.033	0.411	2.600
Other	7	100	0	0	- (b)			
**Job**					**0.401**			
Academic	41	61	26	39		reference category		
Non-academic	43	68	20	32	-	0.733	0.356	1.511
**Religion**					**0.334**			
Religion	38	62	23	38		reference category		
None	41	71	17	29	-	0.685	0.318	1.475

Univariate logistic regression analysis. OR  =  odds ratio; CI  =  confidence interval. (a) Gender, age group, department, employment, ethnicity, job and religion as independent variables (n = 131). (b) No cases with AUDIT score of 8+ so calculations not possible.

Overall, we found no statistically significant differences in socio-demographic characteristics between low risk and hazardous drinkers in our sample. However, when the category ‘Department' was examined, we found that the odds of an employee in central departments being AUDIT positive is only one third of that of an employee in science and health-related departments (OR = 0.35, 95% CI = 0.14–0.91; **Table 3**).

As gender and department were both moderately close to significance, we investigated them simultaneously using logistic regression to see how they related to the cut-off score independent of the effect of each other. Neither gender nor department were significant in the multivariate analysis (**Table 4**).

**Table 4:** Association between drinking status, gender and department.

Characteristic^(a)^	Adjusted p value	OR	95% CI for OR	
			Lower	Upper
**Gender**	**0.084**			
Female		reference category		
Male	-	1.949	0.914	4.157
**Department**	**0.059**			
Science and health-related		reference category		
Arts	0.283	0.556	0.190	1.624
Central departments	0.021	0.322	0.122	0.846

Multivariate logistic regression analysis of gender and department.

(a) Gender and department as independent variables.

### AUDIT total and individual scores

The distribution of the total scores among the respondents is displayed in **Figure 1**. Overall, the mean AUDIT score was 6.7 (standard deviation 4.3). Among men (n = 53), total score ranged from zero to 21 (mean 7.2, median 7.0) and among women (n = 74) from zero to 18 (mean 6.3, median 5.0).

**Figure 2:**
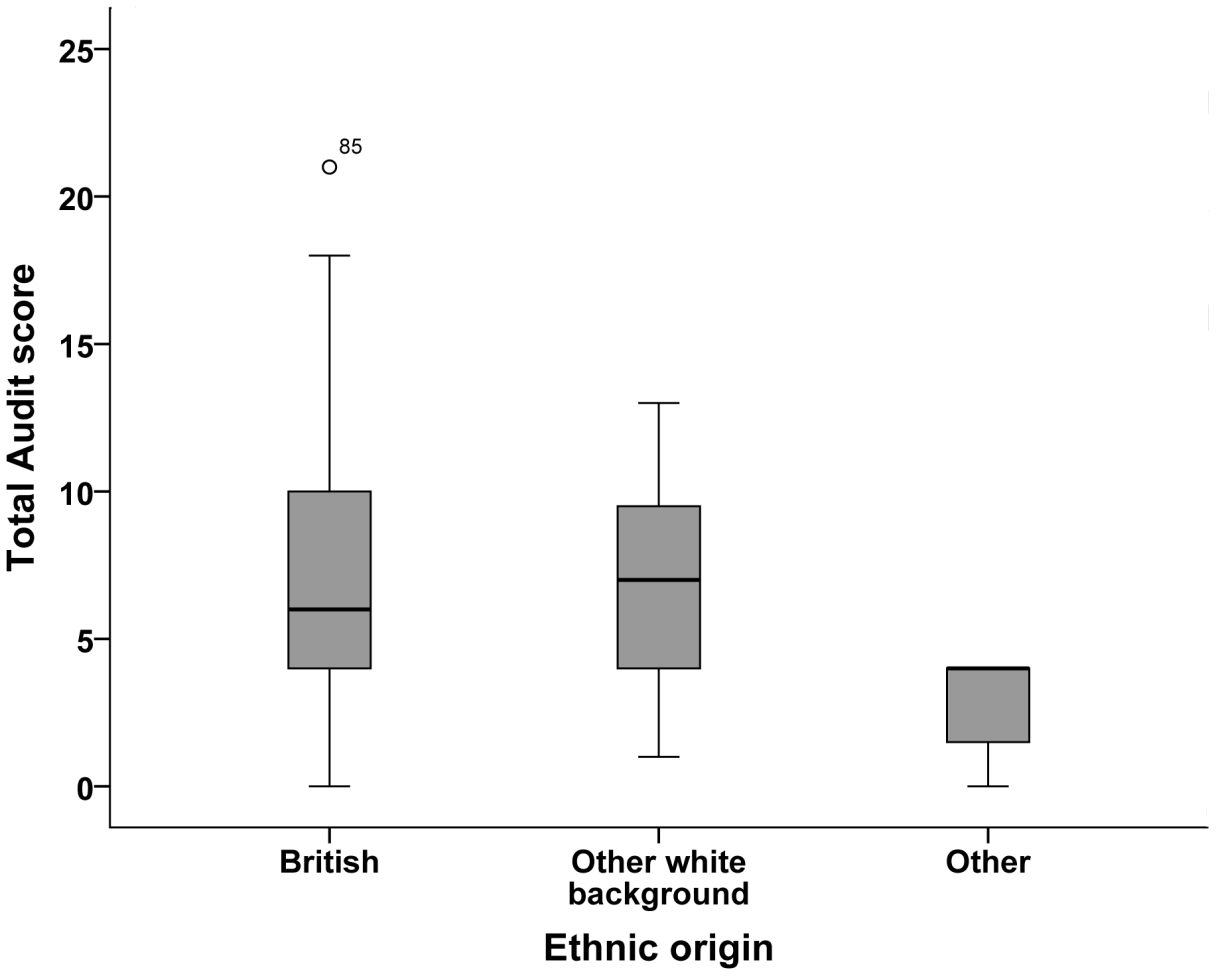
Boxplot of total AUDIT scores by ethnic origin. Kruskal-Wallis test: H = 7.9, df = 2, p = 0.019. The principal difference is that Other is lower (Mann-Whitney test: U = 133, p = 0.007).

Socio-demographic differences in total scores were assessed using the Mann-Whitney test for gender and the Kruskal-Wallis test for the other demographic characteristics. No significant differences were found between demographic categories, with the exception of ethnicity (**Figure 2**: H = 7.9, df = 2, p = 0.019). The principal difference is that ‘Other’ is lower (Mann-Whitney test: U = 133, p = 0.007).

The results and percentage of respondents to each question are displayed in **Tables 5** **and** **6** **.** Ninety eight per cent of respondents reported drinking, with 39 per cent consuming three or more drinks on a typical drinking day (**Table 5**). Fifty nine per cent reported consuming six or more drinks on one occasion (a measure of binge drinking; Matano *et al*, 2002), with 28 per cent reporting consumption of six or more drinks per occasion on a monthly or weekly basis (**Table 5**).

**Table 5:** Percentage of respondents to individual AUDIT questions.

AUDIT question	Respondents	Percentage scoring ≥1
**1. How often do you have a drink containing alcohol?**		98%
(0) Never	2%	
(1) Monthly or less	7%	
(2) 2–4 times/month	15%	
(3) 2–3 times/week	35%	
(4) 4 or more times/week	41%	
Total	131	
**2. How many drinks containing alcohol do you have on a typical day when you are drinking?**		39%
(0) 1 or 2	61%	
(1) 3 or 4	33%	
(2) 5 or 6	5%	
(3) 7 to 9	1%	
Total	128	
**3. How often do you have six or more drinks on one occasion?**		59%
(0) Never	41%	
(1) Less than monthly	31%	
(2) Monthly	22%	
(3) Weekly	7%	
Total	130	
**4. How often during the last year have you found that you were not able to stop drinking once you had started?**		21%
(0) Never	79%	
(1) Less than monthly	14%	
(2) Monthly	6%	
(4) Daily or almost daily	1%	
Total	131	
**5. How often during the last year have you failed to do what was normally expected from you because of drinking?**		19%
(0) Never	81%	
(1) Less than monthly	16%	
(2) Monthly	3%	
Total	131	

The percentage of employees responding to specific AUDIT questions is displayed. Question scores are shown in parentheses next to the responses. The percentage of employees scoring 1 or greater on each question is shown.

**Table 6:** Percentage of respondents to individual AUDIT questions.

AUDIT question	Respondents	Percentage scoring ≥1
**6. How often during the last year have you needed a first drink in the morning to get yourself going after a heavy drinking session?**		1%
(0) Never	99%	
(1) Less than monthly	1%	
Total	131	
**7. How often during the last year have you had a feeling of guilt or remorse after drinking?**		41%
(0) Never	59%	
(1) Less than monthly	34%	
(2) Monthly	6%	
(3) Weekly	2%	
Total	131	
**8. How often during the last year have you been unable to remember what happened the night before because you had been drinking?**		22%
(0) Never	78%	
(1) Less than monthly	19%	
(2) Monthly	4%	
Total	130	
**9. Have you or someone else been injured as a result of your drinking?**		14%
(0) No	86%	
(2) Yes, but not in the last year	12%	
(4) Yes, during the last year	2%	
Total	131	
**10. Has a relative or friend or a doctor or other health worker been concerned about your drinking or suggested you cut down?**		18%
(0) No	82%	
(2) Yes, but not in the last year	8%	
(4) Yes, during the last year	10%	
Total	131	

The percentage of employees responding to specific AUDIT questions is displayed. Question scores are shown in parentheses next to the responses. The percentage of employees scoring 1 or greater on each question is shown.

Only one respondent (1%) reported that they needed a first drink in the morning to get going after a heavy drinking session (**Table 6**). Nineteen per cent of respondents claimed they had failed to do what was normally expected from them in the past year due to drink, although this occurred less than monthly for the majority (16%). Not being able to stop drinking once started during the last year was reported by 21 per cent of the respondents (**Table 5**).

Eight per cent of respondents felt guilt or remorse after drinking at least once within the last month (**Table 6**). Furthermore, 14 per cent of respondents reported that they or someone else had been injured due to their drinking, although the majority of these (12%) did not occur during the last year. Eighteen per cent of respondents reported that someone close to them or a health professional had been concerned about their drinking or suggested they cut down, with 10 per cent reporting that this had occurred during the past year. Finally, 22 per cent of respondents were unable to remember what happened the previous night due to their drinking (**Table 6**).

## Discussion

### Principal findings

This study examined the prevalence of hazardous drinking in 131 employees at a UK university. A high prevalence of hazardous drinking was evident (35%). We believe this is one of the highest levels ever reported. In general, hazardous drinking does not appear to be associated with socio-demographic characteristics. However, departmental differences were observed: the odds of being a hazardous drinker for an employee in science and health-related departments were almost three times that of an employee in central departments. Minority employees had significantly lower total scores than white employees.

There is a lack of readily available data on the prevalence of hazardous drinking in university employees in the UK. The present research therefore contributes to the public health literature by presenting empirical results from a unique data set. Implications from this study include employee safety, alcohol policy formulation and work productivity. A high proportion of employees are at risk of alcohol-related problems. Indeed, some of them are already experiencing alcohol-related harm (22 per cent reported having blackouts after a night of drinking and 14% had either injured someone or been injured themselves due to their alcohol use). Furthermore, 28 per cent of employees reported binge drinking (greater than six drinks in a single session) on a monthly or weekly basis. This pattern of drinking may carry additional health risks [32]. The high prevalence of hazardous drinkers reported in this study is of particular concern, given the type of institution in which they work. For example, a high proportion of staff regularly encounter young adults and are responsible for them while they are in their care. Some operate or supervise the use of expensive and dangerous equipment and procedures. Furthermore, many teaching staff in medical and related departments have dual roles as clinicians and, therefore, are bound by profession and fitness-to-practice regulations. The higher prevalence of hazardous drinking in science and health-related departments may be due to subculture and differences in stress levels and/or responses, and merits further research.

### Strengths and weaknesses of the study

It is important to point out that the AUDIT questionnaire is a universal screening tool used in numerous studies and endorsed by the World Health Organization. The small sample size (n = 131) calls for some caution in interpreting our findings, which may not be representative of university employees. However, in general alcohol misuse is underreported [33]. Furthermore, the response rate of returned questionnaires (77% in this study) was greater than for other professional or educated groups (38–73%). Attempts to increase the initial response rate might have been seen as institutional harassment.

The use of a self-report questionnaire may give rise to response bias, such as under-reporting of alcohol intake, due to fear of stigmatisation or self-denial. This may be more likely to occur in a higher education institution. However, the anonymity of the questionnaire survey may have encouraged participants to respond truthfully. Some respondents may not be aware of how much alcohol they consume (e.g. how many units of alcohol are in a drink). Furthermore, respondents were required to make judgements about their drinking behaviours during the past year, which may have been affected by poor recall. There is also the issue of non-response bias; the characteristics of non-responders could not be obtained because the study was anonymous.

In the present study, the respondents were predominantly drinkers (98%), with over a third (35%) being hazardous drinkers. This finding compares to 90% per cent of people who drink alcohol in England [34]. Similarly, Vasianovich *et al* also found that 90% of male employees at a UK higher education institution consumed alcohol [31]. The prevalence rate of hazardous drinking found in this study compares to almost a quarter of hazardous drinkers (24%) in England [22], 5% in a highly educated workforce (Silicon Valley, California) [30], 17% in UK NHS hospital consultants [29] and 32 per cent in police personnel (Queensland, Australia) [16], also measured using the AUDIT.

The fact that the vast majority of the respondents were drinkers could also introduce issues of bias. However, the extensive number of drinkers in the sample should not be regarded as a major flaw since the focus of the study was to assess the prevalence of hazardous drinking (rather than drinking in general). Overall, the significantly high prevalence of hazardous drinking needs to be considered. It is hoped that this study would stimulate wider discussion of the impact of alcohol misuse in the workplace and, in particular, in academia.

It is reported that socio-demographic predictors of alcohol consumption include age and sex [35]. Our sample included a higher percentage of male (43%) compared to female (30%) hazardous drinkers, although this gender difference was not statistically significant. Similarly, in a national survey the proportion of hazardous drinkers was higher among men (33% of men compared to 16% of women) [22]. Seppa *et al* reported that 31 per cent of male and 11 per cent of female employees at academic institutions in Finland were suspect heavy drinkers, measured using an AUDIT score ≥8 [28]. Similarly, Hermansson *et al* reported a higher percentage of male (13%) than female (6%) AUDIT-positive employees [15]. Notably, the percentage of female hazardous drinkers in the present study is higher than in the other studies, which may be due to subculture or socio-economic differences between the general population and the university employees in our sample.

We did not find any age and ethnicity differences between lower-risk and hazardous drinkers as reported in the general population [22]. This may reflect more homogeneity in a university workforce than in the general population. Curry *et al* did not find any demographic differences between light drinkers and at-risk drinkers in occupational medicine patients [36]. Interestingly, AUDIT total scores of minority employees were significantly lower than white British and other white employees, which were similar. Minority employees scored no higher than four on the AUDIT. However, with so few of them (n = 7), it would be unwise to rely on the results. In a U.S. national survey, Frone reported that minority employees were less likely to be alcohol impaired (i.e. working under the influence of alcohol or with a hangover) in the workplace than white employees [14]. By contrast, Matano *et al* found that problem drinking was more prevalent in African-American employees [30].

The sample contained a high percentage of white (95%) and British (76%) employees. There may have been a relative increase in the number of white and British participants returning the questionnaire but it is difficult to speculate on the reasons for this due to the sample size. Nevertheless, this does not mean that the sample is not reflective of the wider university population with respect to ethnicity. Given that this is an epidemiological study and ethnicity was one of the factors we were investigating, the observation that the higher proportion of white and British employees might be an underlying factor for the high prevalence of ‘heavy drinking’ should not be seen as a source of bias.

### Unanswered questions and future research

The high prevalence of hazardous drinking may perhaps be attributed to factors pertaining to the work environment, such as availability of alcohol, stress and workplace culture, which may facilitate excessive or regular alcohol consumption [12], [37]. This university is located in an inner city where university employees may be under a higher level of stress compared to their counterparts in the outskirts. Some studies have demonstrated significant differences in patterns of consumption between occupations, which suggests that workplace environments may influence employee alcohol consumption differentially [38]. Of course the work needs to consider that numerous factors are modulators of substance misuse such as stress, alienation, cultures and subcultures [37]. However, it was not within the scope of the present study to measure these factors.

Further work is needed to determine the implications of hazardous drinking on work productivity and safety, and the impact of brief intervention in the university setting.
